# Protective Effects of Plant-Derived Compounds Against Traumatic Brain Injury

**DOI:** 10.1007/s12035-024-04030-w

**Published:** 2024-03-01

**Authors:** Danial Khayatan, Seyed Mehrad Razavi, Zahra Najafi Arab, Maryam Khanahmadi, Amirreza Samanian, Saeideh Momtaz, Vasily N. Sukhorukov, Tannaz Jamialahmadi, Amir Hossein Abdolghaffari, George E. Barreto, Amirhossein Sahebkar

**Affiliations:** 1 Department of Toxicology & Pharmacology, Faculty of Pharmacy, Tehran Medical Sciences, Islamic Azad University, Tehran, Iran; 2 GI Pharmacology Interest Group (GPIG), Universal Scientific Education and Research Network (USERN), Tehran, Iran; 3 Department of Neurology, Faculty of Medicine, Tehran Medical Sciences, Islamic Azad University, Tehran, Iran; 4 Medicinal Plants Research Center, Institute of Medicinal Plants, ACECR, Karaj, Iran; 5 Department of Toxicology and Pharmacology, School of Pharmacy, and Toxicology and Diseases Group, Pharmaceutical Sciences Research Center (PSRC), The Institute of Pharmaceutical Sciences (TIPS), Tehran University of Medical Sciences, Tehran, Iran; 6 Institute for Atherosclerosis Research, Osennyaya Street 4-1-207, Moscow, 121609 Russia; 7 Petrovsky National Research Centre of Surgery, Moscow, Russia; 8 Medical Toxicology Research Center, Mashhad University of Medical Sciences, Mashhad, Iran; 9 Pharmaceutical Research Center, Pharmaceutical Technology Institute, Mashhad University of Medical Sciences, Mashhad, Iran; 10 Department of Biological Sciences, University of Limerick, Limerick, Ireland; 11 Biotechnology Research Center, Pharmaceutical Technology Institute, Mashhad University of Medical Sciences, Mashhad, Iran; 12 Applied Biomedical Research Center, Mashhad University of Medical Sciences, Mashhad, Iran

**Keywords:** Traumatic brain injury (TBI), Polyphenol, Neurodegenerative diseases, Neuroinflammation, Oxidative stress

## Abstract

Inflammation in the nervous system is one of the key features of many neurodegenerative diseases. It is increasingly being identified as a critical pathophysiological primitive mechanism associated with chronic neurodegenerative diseases following traumatic brain injury (TBI). Phytochemicals have a wide range of clinical properties due to their antioxidant and anti-inflammatory effects. Currently, there are few drugs available for the treatment of neurodegenerative diseases other than symptomatic relief. Numerous studies have shown that plant-derived compounds, in particular polyphenols, protect against various neurodegenerative diseases and are safe for consumption. Polyphenols exert protective effects on TBI via restoration of nuclear factor kappa B (NF-κB), toll-like receptor-4 (TLR4), and Nod-like receptor family proteins (NLRPs) pathways. In addition, these phytochemicals and their derivatives upregulate the phosphatidylinositol-3-Kinase/Protein Kinase B (PI3K/AKT) and nuclear factor erythroid 2-related factor 2 (Nrf2) pathways, which have critical functions in modulating TBI symptoms. There is supporting evidence that medicinal plants and phytochemicals are protective in different TBI models, though future clinical trials are needed to clarify the precise mechanisms and functions of different polyphenolic compounds in TBI.

## Introduction

There is a growing public health concern associated with traumatic brain injuries (TBIs) in people under 45 years of age. TBI is predicted to become the third most common cause of death worldwide by 2020, according to the World Health Organization (WHO) [1]. It is estimated that patients who survive severe TBI are permanently disabled in terms of neurological and psychological functioning and suffer a heavy financial burden. According to Wittchen et al., the costs associated with TBI exceed €33 billion annually in Europe alone, making TBI a key research area on many national agendas [2]. Despite advances in prevention and early resuscitation techniques, long-term neurological morbidity and overall neurological recovery remain major health challenges. To the best of our knowledge, there are currently no approved treatments for TBI that have been approved by any of the regulatory agencies in order to prove their effectiveness [3]. TBI is complex and heterogeneous, and conventional methods of diagnosing and treating it have proved inadequate. As a consequence, alternative methods for developing TBI therapies should be explored [4].

**Fig. 1:**
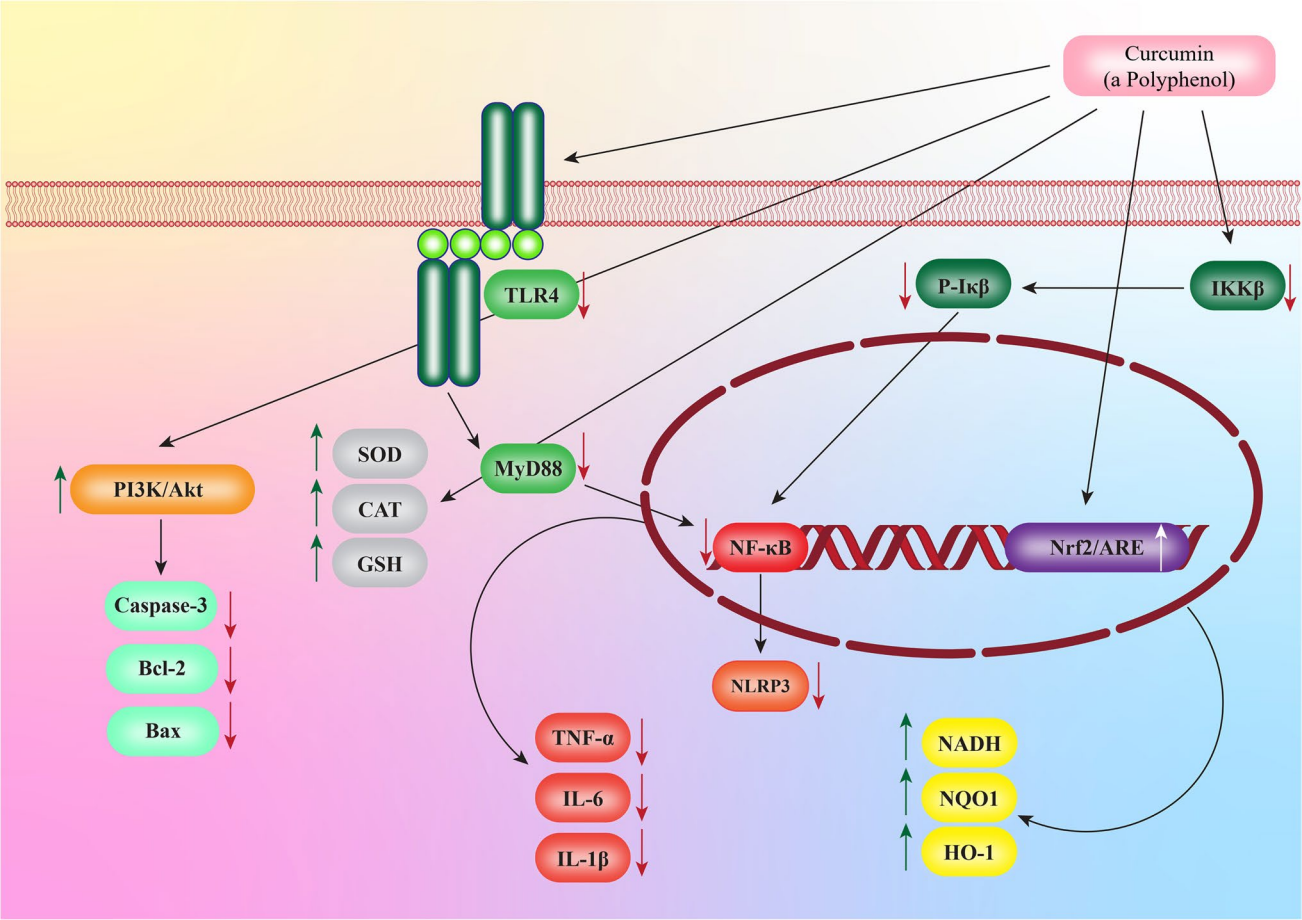
Therapeutic advantages of curcumin, a polyphenol, against traumatic brain injury through interaction with different inflammatory signaling pathways and their effects on levels of cytokines and related biomarkers

Medicinal plant sand their bioactive ingredients, in particular polyphenols, have been implicated in various biological activities, including, but not limited to, immunomodulation, anti-inflammatory, cardiovascular protection, antioxidant, and anticancer potential [5–7]. It is well known that plants produce polyphenols in the form of glycoside esters and free aglycones. The polyphenol family contains more than 8000 structural variants [8]. Fruits and vegetables contain polyphenols, bioactive compounds responsible for their color, flavor, and health benefits. Based on their chemical structures, they can be divided into several different classes: flavonoids such as flavonols, flavones, isoflavones, neoflavonoids, anthocyanidins, chalcones, and proanthocyanidins, phenolic acids, stilbenoids and phenolic amides [9]. Multiple aromatic rings are attached to hydroxyl ligands to form these molecules, which are predominantly plant metabolites. They can be classified according to their chemical structures [10]. Antioxidants derived from polyphenolic compounds are mainly phenolic compounds derived from plants. Some of these compounds may exist as ester derivatives (e.g., epigallocatechin gallate) or ether derivatives (e.g., ferulic acid) [11]. A polyphenolic compound can be divided into two categories based on the type of phenolic compound it belongs to. In addition, these flavonoids have also been found in pharmaceutical preparations. Among those are epigallocatechin gallate, epigallocatechin, catechins, fisetin, luteolin, and quercetin, which are often used as ingredients in pharmaceutical preparations [12]. There is an active ingredient in Quercetin which inhibits the activity of BACE-1, which is a cleaving enzyme of the amyloid precursor protein [13]. In neurodegenerative diseases like Parkinson’s disease (PD), traumatic brain injury (TBI), and Alzheimer’s disease (AD), flavonoids and NSAIDs modulate the nuclear factor-kappa β (NF-κB) signaling pathway [14]. By scavenging free radicals, polyphenols protect against chronic diseases characterized by the involvement of free radicals in their pathogenesis. Some plant species, such as black soybeans, contain polyphenolic phytochemicals that are effective in maintaining human health, particularly in preventing cancer, neurological disorders, cardiovascular disease, and diabetes. However, their effect on the progression of TBI is controversial, perhaps because of the irreversible brain damage following TBI [15]. A review of various in vitro and in vivo studies on the therapeutic benefits of plant-derived phyotchemicals, with curcumin as an example, in TBI has been presented in this review (Fig. 1).

## Pathogenesis of TBI

There has been a rapid increase in TBIs and they are considered a highly complex condition. Accidents involving motor vehicles, abuse, and concussions are among the leading causes of TBI. According to research, there are three levels of severity associated with this disease, mild, moderate, and severe. During recovery, there is sometimes a debilitating and transient neurological disorder. While injuries to other parts of the body contribute significantly to brain damage in severe cases such as polytrauma, injuries to the brain can cause even more significant damage [16].

There are several injuries that can result from TBI, including primary and secondary injuries. Besides lacerations and contusions, primary injuries can result in hemorrhages and ruptures. In addition to the primary injury caused by the traumatic event itself, the secondary injuries that develop as a result of inflammation, oxidative stress, and glutamate toxicity have been shown to play a significant role in determining the outcome of a traumatic brain injury. Edema and cell death occur in the brain due to calcium imbalance and oxidative stress after a post-TBI injury [17]. Often, secondary injury is observed within a few hours to a few days of the initial injury due to a cascade of events. After the primary injury mechanism is triggered, a second wave of pathological changes, commonly known as secondary injury mechanisms, is initiated, including metabolic changes, neuronal inflammation, vascular complications, cell death of glia and neurons, axonal damage, and mitochondrial dysfunction. Damaged mitochondria produce large amounts of oxygen near the site of injury. Autophagy destroys these mitochondria for relative neuronal protection and reduces oxidative stress [18, 19]. In neurons that have been damaged by cell death proteins such as those associated with mitochondrial apoptosis are triggered when mitochondrial membranes are damaged and permeability increases. Autophagy is one of the processes in the starvation response that is regulated by the organ and is responsible for the destruction and recycling of cellular components, as well as participating in organ circulation and controlling bioenergetics in the body [20]. Furthermore, autophagy prevents mitochondrial apoptosis or neuroinflammation, in addition to its neuroprotective effects. Furthermore, it is also important to note that excessive autophagy can also contribute to the generation of neuronal death when there are ischemic or hypoxic conditions, although its inhibition does the opposite.

Some signaling pathways such as phosphatidylinositol 3-kinase/ protein kinase B (PI3K/AKT), are involved in apoptosis, and induction of this pathway can suppress it post-TBI [21]. The secondary injury that results from TBI is characterized by an array of inflammatory responses. The Toll-like Receptors (TLRs) in the human immune system are located on the surface of the cells and are responsible for activating the immune system by releasing endogenous ligands and recognizing a wide range of pathogen-associated molecular patterns (PAMPs), including lipopolysaccharides (LPS) flagellin, and single-stranded and double-stranded viral RNA. Multiple aspects of CNS homeostasis are affected by these patterns, which release enzymes and cytokines that trigger an inflammatory cascade [22]. There is increasing evidence that TLR4 plays a critical role in the initiation of inflammatory responses after trauma. TLRs activate two distinct pathways leading to the activation of transcription factors that regulate the expression of pro-inflammatory cytokine genes. Myeloid differentiation factor 88 (Myd88) activates the TNF receptor-associated factor 6 (TRAF6), producing pro-inflammatory cytokines. There is an interaction between adapter-inducing interferons (TRIF) that contain TIR domains, which are activated in a pathway independent of Myd88, and induce nuclear factor kappa B (NF-κB) to produce inflammatory mediators [23, 24].

The NF-κB family consists of five proteins, including NF-κB1 (p50), NF-κB2 (p52), p65, RELB, and c-REL. Both endogenous and exogenous ligands activate NF-κB and, when activated, is found in neurons and glia under various \inflammatory conditions. In addition to the canonical pathway, a non-canonical pathway activates NF-κB [25]. The NF-κB pathway is also involved in a wide range of immune responses and various cellular progressions such as apoptosis and proliferation. As a result of TBI, activation of NF-κB releases several inflammatory agents that lead to secondary brain damage [26]. A recent study has shown that TBI exacerbates oxidative stress by producing free radicals [27]. There is increasing evidence that oxidative stress plays an important role in secondary damage in relation to primary damage. The nuclear factor erythroid 2 related factor 2 (Nrf2) is involved in a variety of functions within cells and accumulates in their cytoplasm under normal conditions [28]. In addition to regulating genes involved in oxidative stress, Nrf2 acts as a transcription factor. As a result of this component of Nrf2, antioxidant enzymes such as malondialdehyde (MDA), heme oxygenase-1 (HO1), superoxide dismutase (SOD), quinone oxidoreductase-1 (NQO1), and glutathione peroxidase (GSH-Px) are activated [29]. This enzyme acts by directly regulating the levels of reactive oxygen species (ROS), meaning that it can directly react with free radicals without harming the cells' vital function. The antioxidant response element (ARE) found in the gene promoter allows Nrf2 to promote the expression of antioxidant genes. The Nrf2/ARE signaling pathway modulates several pathological processes, including oxidative stress [29, 30].

## Effect of Different Phytochemicals and Polyphenols on TBI Through Inflammatory Signaling Pathways

Polyphenols exert antioxidant activity through various mechanisms, some of which are multi-step mechanisms [31]. However, free radical scavenging is widely recognized as one mechanism by which polyphenols exert their antioxidant effects. The extended conjugation of the unpaired electron is another key property of polyphenolic antioxidant radicals that contributes to their increased stability. By quenching the reactive nitrogen species (RNS) and reactive oxygen species (ROS) produced by free radicals as they pass through a polyphenolic compound, polyphenolic compounds manifest their antioxidant benefits [32].

As a result of free radical oxidation, ROS, RNS, and other compounds are produced. The ROS and RNS commonly observed in free radical-induced oxidative damage include nitric oxide (·NO), hydroxyl radicals (·OH), superoxide radicals (O_2_^–^), and peroxynitrite anion (O = NOO^–^). Fenton reactions catalyzed by metal ions (e.g., Fe^2+^ and Cu^+^) generate ROS and RNS. When ROS and RNS are subjected to high levels of oxidative stress, they are highly reactive and have short half-lives, which, in conjunction with an imbalance between their production and destruction, can cause a significant amount of protein modifications as well as other reaction products to accumulate at the site of inflammation as a result of ROS and RNS-derived proteins. Inflammatory conditions such as cancer, atherosclerosis, AD, and traumatic brain injury may arise as a result of an excessive accumulation of ROS and RNS-derived reaction products [33–35]. In addition to polyphenols, citrus flavanones, including hesperetin, hesperidin, and neohesperidin, can cross the blood–brain barrier (BBB). BBB permeability varies between polyphenols. In addition, animal studies has shown that blueberry consumption induces anthocyanin accumulation in the cortex and cerebellum of animal models (rats and pigs) [36]. Furthermore, the valerolactones undergo further metabolism resulting in the formation of phenolics and polyphenols, such as (hydroxyaryl)propanoic acid, (hydroxyaryl) cinmmic acid, (hydroxyaryl)valeric acid, hydroxybenzoic acid, and (hydroxyaryl)acetic acid derivatives. Secondary polyphenolic metabolites have a relatively higher degree of bioavailability and are more permeable to the BBB than flavonoids or dietary flavonoids and may inhibit neuroinflammation. As a result, polyphenols can be used therapeutically in a wide range of neurodegenerative diseases as well as TBI (Table 1 and 2) [37, 38].

**Table 1:** In vivo interventions

Study design	Disease	Intervention		Number of animals		Treatment duration	Results	Ref
		Case	Control	Case	Control			
**Male SD rats**	TBI	Cur (10 mg/kg, i.p Cur (20 mg/kg, i.p.); Cur (30 mg/kg, i.p.); Cur (50 mg/kg, i.p.);	Vehicle	*n* = 5	*n* = 5	5 weeks	1) ↓ Levels of NLRP3, IL-1β, IL-6, IL-18, TNF-α 2) ↑ BDNF/TrkB and PI3K/Akt signaling	[52]
**Male SD rats**	FPI-induced TBI	FPI + Cur derivatives (500 ppm, oral); Cur derivatives (500 ppm, oral); FPI-regular diet	Regular dietSham	*n* = 6–8	*n* = 6–8	1 week	1) ↓ SOD, Sirtuin 2 2) ↓ BDNF 3) ↓ CREB 4) ↓ Synapsin I	[54]
**Wild type and** **Nrf2-knockout** **C57BL/6 mice**	TBI (weight-drop impact)	TBI + Cur (500 mg/ kg/day, i.p.); TBI + vehicle (DMSO equal volume, i.p.);	No treatment	*n* = 6–8	*n* = 6–8	1 day	1) ↑ Nrf2 expression & Activation 2) ↓ Ipsilateral cortex injury 3) ↓ Neutrophil infiltration 4) ↓ Microglia activation	[55]
**Male SD rats**	LFP-induced TBI	TBI + Cur (50 mg/kg, s.c.); TBI + SAHA (50 mg/ kg, i.p.);	Vehicle (50 mg/kg, i.p.)	*n* = 6	*n* = 6	2 weeks	1) ↓ HAT 2) ↓ Mechanical sensitization	[56]
**Male ICR mice**	TBI	TBI TBI + Vehicle (equal volume of 10% DMSO, i.p.); TBI + Cur (50 mg/kg, i.p.); TBI + Cur (100 mg/ kg, i.p.);	No treatment	*n* = 6	*n* = 6	3 days	1) ↑ Nrf2 expression & activation 2) ↓ Bcl-2 3) ↓ Caspase-3 4) ↑ HO-1, NQO1, and NAD(P)H 5) ↑ GPx and SOD 6) ↓ MDA	[57]
**Male SD rats**	FPI-induced TBI	FPI + Cur derivatives (500 ppm, oral); Cur derivatives (500 ppm, oral); FPI-regular diet;	Regular diet + Sham	*n* = 6	*n* = 6	4 weeks	1) ↓ BDNF 2) ↓CREB 3) ↓ Synapsin I	[58]
**Male Wistar rats**	Subventricular zone surgery-induced TBI	TBI + Cur (100 mg/ kg, i.p.); TBI + Cur + PM (100 mg/kg, i.p.); TBI + Cur + NS/PCs (100 mg/kg, i.p.); TBI + Cur + PM + NS/PCs (100 mg/kg, i.p.);	DMSO	*n* = 6	*n* = 6	4 weeks	Cur + PM + NS/PCs can also: 1) ↓ Lesion size, astrogliosis, macrophage, and microglial reaction 2) ↓GFAP 3) ↓ Iba1 and CD68	[59]
**Male SD rats**	TBI (weight-drop impact)	Sham; TBI; TBI + THC (10 mg/ kg, i.p.); TBI + THC (25 mg/ kg, i.p.); TBI + THC (50 mg/ kg, i.p.);	Vehicle (saline containing 1% DMSO)	*n* = 12	*n* = 12	1 week	1) ↑ Nrf2 expression & activation 2) ↓ Bcl-2 3) ↓ Bax 4) ↓ Caspase-3 5) ↑ HO-1, NQO1, and NAD(P)H 6) ↑ GPx and SOD 7) ↓ MDA	[60]
**Male SD rats**	FPI-induced TBI	FPI + Cur (500 ppm, oral); FPI + DHA + Cur (1.2%, 500 ppm, oral); FPI + regular diet; FPI + DHA diet (1.2 %, oral);	Regular diet + Sham	*n* = 6	*n* = 6	2 weeks	FPI-DHA-Cur can also; 1) ↓ BDNF and its receptor p-trkB 2) ↓ Stress oxidative damage 3) ↓ Plasticity markers	[61]
**Male SD rats**	TBI	TBI; TBI + vehicle (saline containing 1% DMSO, i.p.); TBI + THC (25 mg/ kg/day, i.p.); TBI + THC (50 mg/ kg/day, i.p.);	No treatment	*n* = 6	*n* = 6	3 days	1) ↓ Brain edema 2) ↓ Apoptosis and neuron cell death 3) ↑Neurobehavioral function 4) ↑ p-AKT 5) ↑ activation of PI3K/AKT	[63]
**SD rats**	TBI	Quercetin (50 mg/kg, i.p.) (0.5, 12, and 24 h after TBI)	-	*n* = 10	*n* = 10	3 days	1) cortical Nrf2/HO-1 pathway activation 2) improvement in neuroinflammation and oxidative stress	[88]
**Female SD rats**	TBI (weight-drop injury)	Quercetin (30 mg/kg,i.p.) (0, 24, 48, and 72 h post TBI)	Saline	*n* = 15	*n* = 15	4 weeks	1) ↑ cognitive function 2) ↓ oxidative stress 3) ↑GSH-Px, SOD, and CAT activity	[90]
**Male WS rats**	TBI (weight-drop impact)	1) Quercetin (50 mg/kg,i.v.) 2) Mannitol (1 mg/kg, i.v.)	-	*n* = 10	*n* = 10	4 h	1) ↓MDA levels 2) ↓EPO Serum levels by mannitol 3) quercetin would be used as an alternative treatment	[89]
**Male SD rats**	TBI (weight-drop impact)	1) Quercetin (50 mg/kg, i.p.) (0.5, 12 h, and 24 h after TBI) 2) Quercetin (50 mg/kg, i.p.) (0.5, 12 h, and 24 h after TBI) + LY294002 10 μL(50 mM in 25% dimethyl sulfoxide in PBS (injection into the left ventricle))	-	*n* = 25	*n* = 25	9 days	1)improvement in neurological impairment and cognitive function 2) ↓ neuronal autophagy and apoptosis via PI3K/Akt activation	[91]
**Male SD rats**	TBI (weight-drop impact)	Quercetin (50 mg/kg, i.p.) (0.5, 12, and 24 h after TBI)	-	*n* = 25	*n* = 25	5 days	1) inhibition of ERK1/2 and PI3K/Akt activation 2) improvement in brain edema and motor functions	[92]
**Male ICR mice**	TBI (weight-drop impact)	Quercetin (50 mg/kg, i.p.) (30 min after TBI)	DMSO + 0.9% saline	*n* = 24	*n* = 24	24 h	1) ↑PGC-1α 2) ↓cytochrome c, MDA, and SOD levels	[93]
**Male ICR mice**	TBI (weight-drop impact)	Quercetin (50 mg/kg, i.p.) (30 min after TBI)	DMSO + 0.9% saline	*n* = 30	*n* = 30	24 h	1) ↑ expression and activity of antioxidant enzymes 2) Nrf2 pathway activation	[94]
**Male ICR mice**	TBI (weight-drop impact)	Quercetin (50 mg/kg, i.p.) (30 min after TBI)	Vehicle (DMSO + 0.9% saline)	*n* = 24	*n* = 24	24 h	1)↑ PGC-1α expression 2)↑ mitochondrial function 3)↓ cytochrome c, MDA, and SOD	[112]
**WS rats**	TBI (weight-drop impact)	Quercetin (10 mg/kg/ day, i.p.)	-	*n* = 10	*n* = 10	7 days	1)↓ oxidative-nitrosative stress 2)↓ peroxynitrite concentration 3)↓ iNOS activity	[113]
**Male SD rats**	TBI (CCI)	Catechin ( 1, 5, 10, 20, 30 mg/kg/ day, o.g.)	0.5% DMSO (1 mg/kg/day, o.g.)	*n* = 10	*n* = 10	28 days	1↓tight junction disruption 2) ↓BBB disruption 3) neuroprotective	[96]
**Male C57BL/6 mice**	TBI (CCI)	1)EGCG (0.2%, w/v) in drinking water immediately after TBI 2) AICAR (500 mg/kg, i.p.) (5 min and 24 after TBI 3) Metformin (250 mg/kg, i.p.) (5 min and 24 after TBI)	Normal drinking water	*n* = 16	*n* = 16	18 days	1) ↑AMPK pathway 2) preventing locomotor and cognitive damage 3)↓phosphorylation of IKKα/β and IκBα	[97]
**Male WS rats**	TBI	EGCG (0.1%, w/v) In drinking water	Normal drinking water	*n* = 27	*n* = 27	7 days	1)↓ O2¯ and OH¯ 2)improvement in cerebral function 3)↓ neuronal degeneration and apoptotic cell death	[98]
**Male WS rats**	TBI (CCI)	EGCG (0.1% w/v) in drinking water	Normal drinking water	*n* = 18	*n* = 18	7 days	1) protection of nestin-positive cells, including NSCs 2)↓ free radical production’s cell death of neuronal cells and NSCs	[99]
**Male WS rats**	TBI	1) EGCG (0.1% w/v) in drinking water pre-TBI 2) EGCG (0.1% w/v) in drinking water pre- and post-TBI 3) EGCG (0.1% w/v) in drinking water post-TBI	Normal drinking water	*n* = 6	*n* = 6	7 days	1)) ↓ O2¯ and OH¯ 2)improvement in cognitive impairment 3) improvement in cerebral function	[114]
**Male SD rats**	TBI (CCI)	Honokiol (1.0 mg/kg/day, i.v.) (8,16 h after TBI)	(2.5% mixture of ethanol and cremophor EL in 5% dextrose)	*n* = 24	*n* = 24	21 days	1) neuroprotective 2)↓ overactive cell cycle 3)↓ cyclin D1, E2F1, and apoptosis in neurons	[107]
**Male C57BL/6 mice**	TBI (CCI)	Sesamin (30 mg/kg/day, i.p.)	vehicle	*n* = 5	*n* = 5	3 days	1)↓ BBB disruption 2) ECs protection 3) anti-oxidative and anti-apoptotic effects on endothelial cells	[101]
**Male SD rats**	TBI	Magnolol (2 mg/kg, i.v.)	CMC (3 cc, i.v.)	*n* = 8	*n* = 8	3 days	1)↓ oxidative brain injury, apoptosis, and neurologic deficits 2) free radical scavenging capability 3) ↑TGF-β1 expression → antineuronal apoptosis	[103]
**SD rats**	TBI (weight-drop impact)	Honokiol (5 mg/kg/day, i.p.)	Isotonic saline solution (5 mg/kg, i.p.)	*n* = 9	*n* = 9	7 days	1) ↓lipid peroxidation 3)improvement in the vascular wall 2) ↓BBB disruption	[106]
**Male C57BL/6 mice**	TBI (cold trauma model)	Cinnamon (10 mg/kg, i.p.) (0.5 h after TBI)	DMSO + 0.9% saline (i.p)	*n* = 9	*n* = 9	24 h	1) anti-inflammatory and antioxidant effects 2) balancing NF-κB, Nrf2, GFAP and inflammatory cytokines 3) neuroprotective	[110]
**Male ICR mice**	TBI (weight drop head trauma device)	Cinnamon (100 μg/mL, p.o.)	Normal Drinking water	*n* = 13–21	*n* = 10	35 days	1) ineffectiveness on anxiety levels and motor activity 2)improvement of cognitive function 3)↓memory loss	[115]
**Male C57BL6 mice**	TBI (CCI)	Sodium benzoate (50 mg/kg/day, p.o.)	NaFO (50 mg/kg/day p.o)	*n* = 6	*n* = 6	26 days	1) neuroprotective 2)↓ iNOS expression 3)↓ vascular damage and size cavity lesion	[111]
**Male SD rats**	TBI (CCI)	RVS (100 mg/kg/day, i.p.) (for 3 days)	Ethanol (2%, i.p.)	*n* = 60	*n* = 60	5 days	1)↓ TLR4/NF-κB signaling pathway 2)↓ brain edema 3)↑ motor and cognitive function recovery 4)↓ neuronal autophagy	[75]
**Male SD rats**	TBI (0310 Impactor)	RVS (100 mg/kg)	Vehicle	*n* = 3	*n* = 3	48 h	1)↓ GSK-3 β -mediated autophagy and apoptosis 2) ROS/GSK-3 β/mitochondria pathway is the cause of cell death	[74]
**Male SD rats**	TBI (CCI)	1) RVS (10, 50 mg/kg, i.p.)	Vehicle	*n* = 10	*n* = 10	21 days	1)↓ contusion volume 2) vulnerable neurons protection in CA1 and CA3 regions preservation of hippocamp 3) beneficial motor and cognitive effects	[76]
**WS rats**	TBI	RVS (100 mg/kg, i.p.) single dose	Saline, including 2% ethanol	*n* = 7	*n* = 7	17 days	1) ↓ anxiety 2) ↑ cortex/hippocampus-dependent memory 3) improvement in cognitive impairment	[83]
**Male C57/Bl6 mice**	TBI (CCI)	RVS (100 mg/kg, s.c.) (5 min and 12 h after TBI	Vehicle (corn oil) (s.c.)	*n* = 10	*n* = 10	3 days	1)↓ neuroinflammation 2)↓ IL-6 and IL-12	[82]
**Female WS albino rats**	TBI (FFW)	RVS (50,100 mg/kg/day, i.p.)	-	*n* = 7	*n* = 7	8 days	1)↑ GSH-Px and SOD 2)↓ MDA and 8-OHdG accumulation	[81]
**SD rats**	TBI	1) RSV (0.2% v/v, p.o.) 2) p38 inhibitor SB203580 (1 mg/kg/day, i.p.)	-	-	-	7 days	1)↓ cognitive deficits, brain apoptosis, and ROS generation 2)↑ p38/Nrf2/HO1 signaling pathway	[80]
**Male SD rats**	TBI (free-falling impact)	1) RVS (100 mg/kg, i.p.) (0.5 h before TBI) 2) Sirtinol (10 mg/kg, i.p.) (0.5 h before TBI)	Saline (2% ethanol content)	*n* = 30	*n* = 6	48 h	1)↓ ROS production and NLRP3 activation 2) Anti-inflammatory effects 3)↑SIRT1	[79]
**SD rats**	mild TBI	1) RVS (50 mg/L) in drinking water 2) 3S supplement (100 g prebiotic, 300 g DHA, and 600 g of standard diet)	95% ethanol (1 ml/L) added to drinking water	*n* = 32	*n* = 14	14 days	1) improvement in neural repair 2) improvement in behavioral deficits 3)↓ Aqp4, Gfap Igf1, NFL, and SIRT1 expression	[78]
**Male SD rats**	TBI (weight-drop impact)	RSV (100 mg/kg/day, i.p.) (up to 5 days)	ethanol (2%)	*n* = 60	*n* = 60	5 days	1)↓ brain edema and neuronal autophagy 2)↑ cognitive functional recovery 3)↑ synaptic proteins	[77]

**Table 2:** In vitro interventions

Study design	Disease	Intervention		Number of cells		Treatment duration	Results	Ref
		Case	Control	Case	Control			
**Cortical astrocytes**	TBI (OGD)	RSV (5, 10, and 25 μM)	Saline	*-*		24 h	1)↓ ERK1/2, and p38MAPK 2) enhancement of ammonia, ischemia, and cell swelling	[73]
**CGNs**	TBI	RSV (5 and 10 μM)	PQQ (1 μM)	380000 380000		4 days	1)↑ number of viable CGNS 2)↑ survival of CGNs in the K + /FCS deprivation model	[72]
**TLR4-/-** **male C57BL/6** **mice**	LPS-induced TBI	Cur (0.5, 1, 2, 5 and 10 μM)	DMSO	300,000		3 days	1) ↑ TLR4, MyD88, and NF-κB 2) ↓ Neuronal apoptosis by ↓ caspase-3 3) ↓ Inflammatory mediator release (IL-1β, IL-6, TNF-α, MCP-1 and RANTES)	[53]

### Curcumin

The recent findings on curcumin demonstrate its remarkable versatility as a molecule that interacts with a variety of molecular targets [39–48]. There are curcumin compounds in the rhizomes of the turmeric plant (*Curcuma longa*), a plant belonging to the Zingiberaceae family. As an antioxidant, anti-infection, anti-inflammatory, and anti-tumor compound, curcumin has been approved by the United States Food and Drug Administration as a safe compound [49]. Various CNS disease models have been shown to be susceptible to curcumin's anti-inflammatory properties, including intracerebral hemorrhage, global brain ischemia, and neurodegeneration, all of which are associated with the inflammation of the CNS. Similarly, curcumin exerts neuroprotective effects in mammals when it crosses the blood–brain barrier. An experiment in mice modeling spinal and bulbar muscular atrophy revealed that 5-hydroxy-1,7-bis (3,4-dimethoxyphenyl)-1,4,6-heptatrien-3-one inhibited the aggregation of pathogenic androgen receptors. Curcumin is suspected to possess neuroprotective properties, but few studies have explored this possibility [49]. Curcumin has been shown to reduce cerebral edema, enhance membrane and energy homeostasis, and influence synaptic plasticity following TBI in a few studies. However, curcumin does not appear to have any immunomodulatory properties on inflammatory reactions.

Curcumin has been shown by numerous studies to be effective in reducing inflammation [50, 51]. A lot of research has shown that curcumin suppresses the activation of NF-κB by inhibiting the phosphorylation and degradation of IκB; as a result, curcumin reduces the inflammation caused by NF-κB. The effects of curcumin have been shown to be not only reduced post-TBI neuroinflammation, but also decreased levels of inflammatory mediators that are produced following a TBI [52]. The anti-inflammatory effects of curcumin were demonstrated in an in vitro study using 100 mg/kg of curcumin [53]. According to the report, curcumin was able to reduce the amounts of damage after TBI induction and apoptosis, particularly in the cortical cell model derived from embryonic 15-day pregnant mice, resulting in reduced damage. One study in rats with TBI administered 30 and 50 mg/kg of curcumin daily for 35 days reduced levels of NLRP3, IL-1β, IL-6, IL-18, and TNF-α [52]. Additionally, PI3K/AKT signaling pathways were activated, whereas the BDNF/TrkB signaling pathway was downregulated. There was a conclusion reached by researchers that if you want to receive the maximum benefits from curcumin, 30 mg per kilogram is the most effective dose. There was a reduction in neuroinflammation and subsequent complications of TBI when curcumin was administered. In another FPI (Fluid percussion injury) rat model, animals received 500 ppm of synthetic curcumin, which penetrated the blood–brain barrier, exhibited improved cognitive and locomotor functioning, as well as decreased SOD, Sirtuin 2, BDNF, and CREB levels [54]. Furthermore, curcumin (at 500 mg/kg/day) decreased injury in the ipsilateral cortex as a result of its ability to enhance neutrophil infiltration (weight drop model-TBI). Furthermore, curcumin reduced apoptosis in cells and increased antioxidant activity [55], suggesting potential neuroprotective effects. The pain levels decreased when curcumin (50 mg/kg) was administered to surgically induced TBI rats [56]. However, the compound did not improve mechanical deficits. However, the compound did not improve mechanical deficits.

Curcumin has been found to inhibit non-selective histone acetyltransferases, suggesting potential benefits through histone acetylation. In a rat model of TBI induced by FPI, curcumin (100 mg/kg) exerted neuroprotective properties by activating the Nrf2 pathway. HO-1, NQO1, GSH-Px, and SOD activity were enhanced, and caspase-3 activity was decreased, while Bcl-2 levels increased, suppressing apoptosis [57]. BDNF, synapsin I, and CREB levels were reduced in animal models following TBI after curcumin treatment (500 ppm) [58]. A combination of curcumin (100 mg/kg) neural stem/progenitor cells and PuraMatrix decreased the size of the lesion cells and their apoptosis. In addition, bromodeoxyuridine, GFAP (Glial fibrillary acidic protein), Doublecortin microtubule-associated protein 2, oligodendrocyte transcription factor, Iba1, and CD68 levels were reduced [59]. According to another study, tetrahydrocurcumin (THC), a primary reduced metabolite of curcumin, when administered at a dose of 500 mcg in a rat model of TBI, reduced neuronal damage associated with the injury. The activities of Nrf2, SOD, and GSH-Px were increased while Bcl-2, Bax, and caspase-3 were reduced by THC [60]. When given oral doses of 100 mg/kg of curcumin per day, oxidative damage was decreased, and omega-3 fatty acid DHA levels and 4-HNE (an indicator of membrane lipid peroxidation) increased [61]. Curcumin supplements may have neuroprotective effects after brain injury and may increase the activity of docosahexaenoic acid and fatty acid-transport protein. According to Sharma et al.'s study published in 2010, curcumin reduced iPLA2, 4-HNE, and STX-3, which improved learning progress and relieved complications associated with TBI [62]. The PI3K/AKT pathway was activated by THC in animal models of TBI according to Gao et al. in 2016. A combination of THC and PI3K/AKT, administered for 72 h, promotes neuroprotection and suppresses apoptosis [63].

### Resveratrol

Resveratrol is a stilbenoid polyphenol with anti-apoptotic, anti-inflammatory, neuroprotective, antidepressant, and antioxidant properties [64–67] despite some controversies on its clinical efficacy [68–71]. It is found in *Polygonum cuspidatum* and other natural sources such as grapes, cranberries, plums, peanuts, and mulberries [64]. Shanan et al. conducted in-vitro research on cerebellar granule neuron cultures. It was shown that resveratrol (RSV) at a dose of 5 μM tended to have wound healing effects, but it was not remarkable. However, it was reported that RSV in combination with pyrroloquinoline quinone (PQQ) did not have synergistic effects on the wound healing process. It was also shown that RSV at doses of 5 and 10 μM could increase the number of cerebellar granular neurons but did not result in a significant increase in neurite length. Furthermore, both RSV and PQQ alone were shown to increase cerebellar granule neuron viability in a fetal calf serum (K + /FCS) model [72]. Taherian et al. settled down a study to investigate the effects of RSV on astrocyte swelling following ammonia exposure, ischemia, and trauma in an in vitro model. It was shown that RSV at the doses of 10 and 25 μM could reduce ammonia-induced astrocyte swelling before or after treatment. In addition, Taherian and colleagues showed that RSV (25 μM) could also reduce astrocyte swelling following trauma as a pre-treatment. In addition, it was observed that 25 μM of this chemical could reduce astrocyte swelling following ischemia as a pre- or post-treatment [73].

In another in vitro study, Len and colleagues showed that RSV could increase neuronal cell survival and simultaneously decrease ROS deposition and mitochondrial damage via downregulation of the glycogen synthase kinase 3 Beta (GSK-3β) pathway [74]. Feng et al. showed that RSV at a dose of 100 mg/kg reduces autophagy in brain cells after TBI occurrence in a rat model. RSV decreased brain edema and neuronal injury while increasing learning and memory performance. It was also observed that RSV decreases LC3-positive NeuN cells, which represent the level of autophagy in neuronal cells. In addition, RSV was shown to exert its beneficial neuroprotective effects via downregulation of NF-κB and TLR4 pathways suppression [75]. Singleton et al. conducted a research to evaluate the effects of RSV on the TBI rat model post-injury. RSV at 100 mg/kg was found to improve motor function and memory ability. The compound reduced neuronal loss (CA1 and CA3) and attenuated behavioral impairment [76]. In 2016, an in vivo study conducted by Feng et al. investigated the neuroprotective effects of RSV. RSV (100 mg/kg) increased synaptic activity and decreased neuronal autophagy, which reduces Beclin 1 and LC3II proteins. RSV also attenuated the brain edema measured by the Morris water maze test and improved neurological impairment and cognition after TBI [77]. In another study, RSV in an adjunctive regimen improves neurological function and reduces the long-term adverse effects of TBI. In a rat model, RSV improves behavioral functions assessed by the beam walk, open field, and elevated plus maze tests in a rat model [78]. According to Zou et al., RSV was found to have neuroprotective effects on TBI after traumatic brain injury. There is evidence to suggest that RSV reduces ROS and inflammation-related cytokines such as interleukin-1β (IL-1β), and IL-18. NLRP3 inflammasome and Sirtuin 1 (SIRT1) activation are affected by RSV, which alleviates brain edema and improves mental function [79].

In 2018, Shi et al. demonstrated that RSV could increase cognitive function, which was assessed by the Morris water. Although it was suggested that RSV administration upregulates the Nrf2, and HO-1 expressions. RSV reduced ROS production and neuronal apoptosis. Furthermore, RSV was shown to exert its beneficial effects via activation of the p38 pathway activation [80]. RSV at the doses of 50 mg/kg and 100 mg/kg reduced neurodegeneration and apoptosis. SOD and glutathione levels increased inversely following RSV administration, while MDA and 8-hydroxy-2′-deoxyguanosine (8-oHdG) levels were decreased [81]. In another in vivo study, RSV at 100 mg/kg suppressed inflammation after TBI by decreasing microglial activation and reducing IL-6 and IL-12 [82]. Sonmez et al. showed that RSV at 100 mg/kg reduced neuronal apoptosis and anxiety after TBI, while improving memory and behavioral function [83].

### Quercetin

There are a variety of pharmacological benefits associated with quercetin (QUE), a natural bioflavonoid found in a wide range of plant species [84–86]. QUE is readily absorbed from the gastrointestinal tract and excreted in the urine via the kidneys. QUE has beneficial effects including antioxidant activity, 5-HT, anti-inflammatory effects, antidepressant properties, and reduction of HPA activation [87]. In Song et al.’s study, QUE at 5, 20, and 50 mg/kg significantly reduced microglia inflammation, inflammatory and inflammatory biomarkers, such as TNF-α, inducible nitric oxide synthase (iNOS), IL-1β, and IL-6. In addition, MDA levels were decreased by QUE as well as catalase (CAT), SOD, and GSH levels were increased by QUE. Additionally, this compound upregulated the Nrf2/HO-1 pathway, which is implicated in its beneficial effects. Additionally, QUE decreased Iba-1 protein levels and brain water levels [88].

Similar results were obtained after the use of QUE (50 mg/kg intravenously). QUE decreased MDA levels with concomitant increases in serum GSH and CAT levels [89]. Another study showed that QUE improved cognitive function after TBI injury. QUE at a dose of 30 mg/kg decreased the IL-6, TNF-α, and IL-1β levels, which led to a reduction in neuroinflammation. QUE also increased the expression of SOD, CAT, GSH, and IL-10 [90]. Similarly, in 2016, Du et al. demonstrated that QUE improves cognitive behavior after TBI via upregulation of the PI3K/AKT pathway. It was elucidated that 50 mg/kg QUE decreased LC-3 positive cells and neuronal apoptosis, probably by reducing caspase-3 and BCL2-associated X (Bax) and inversely increased B-cell lymphoma 2 (Bcl-2) and P-Akt expression [91].

In another in vivo study, QUE at the dose of 50 mg/kg reduced the brain water and cerebral edema. QUE improved motor function and neuronal activity, reducing neuronal apoptosis via activation of the Akt pathway and attenuating the extracellular signal-regulated kinase (ERK 1/2) signaling pathway [92]. In a similar study, QUE attenuated brain edema and neuronal apoptosis via regulation of peroxisome proliferator-activated receptor-γ (PPAR-γ) coactivator-1α (PGC-1α). QUE at 50 mg/kg regulated SOD and MDA levels, reduced oxidative stress, and downregulated caspase-3 expression [93]. According to Li et al., QUE at 50 mg/kg decreased the levels of MDA in rats and increased the levels of SOD. TBI was alleviated, and mitochondrial membrane potential (MMP) increased due to Nrf2 upregulation. [94].

### Catechin

The group of flavonoids found in tea is largely responsible for producing catechins (flavan-3-ol). Among the compounds that can contribute to breast cancer prevention and treatment include (-)-epigallocatechin-3-gallate (EGCG), (-)-epicatechin-3-gallate, and (-)-epigallocatechin. Unfermented green tea is one of the best sources of catechins. There are a variety of types and origins of green tea leaves that have different antioxidant properties. Wine, berries, coffee, and tea also contain catechins naturally. Including catechin-containing products in the diet is recommended because catechins have many health-promoting properties. The most important properties of the catechins are their antioxidant, anti-inflammatory and chemopreventive effects [95]. Jiang et al. conducted important research on the neuroprotective effects of catechin on a TBI rat model. Catechin reduces neuronal damage while improving motor function and cognition. Catechin at 20 mg/kg attenuated brain edema and inflammation, reducing inflammatory cytokines such as IL-1β, iNOS, and IL-6 while increasing the arginase 1 levels (an inflammatory marker). Moreover, the chemical was also beneficial to maintaining the integrity of the blood–brain barrier (BBB) and reducing the loss of some tight junction proteins, such as occludin and zonula occludens protein-1 [96].

In another study in 2020, EGCG showed beneficial effects in improving neurological deficits after TBI in a rat model. EGCG increased learning and memory after administration. It has been claimed that EGCG reduces the expression of I kappa B kinase alpha/beta (IKK α/β), and NF-κB, reducing inflammatory cytokines and oxidative stress [97]. In 2011, Itoh et al. demonstrated that EGCG ameliorated neuronal dysfunction and regulated serum MDA levels in a rat model. The compound also reduced apoptosis and oxidative stress while increasing neuronal survival. Furthermore, the single-stranded DNA-positive cells were alleviated after consumption of EGCG (0.1% w/v) [98]. In another study by Itoh and colleagues, EGCG (0.1% w/v) reduced peroxidation levels and increased the amount of nestin-positive in the injured area after TBI [99].

### Lignans

Lignans are a class of natural antioxidant polyphenols found in whole grains, nuts, beans, seeds, and vegetables. Several neuropathological conditions and neurodegenerative diseases, such as PD and AD, are being studied using plant metabolites as potential therapeutics. Some lignans have been shown to have significant anti-inflammatory, neuroprotective, antioxidant, and immunomodulatory properties [100]. Liu et al. in 2017 investigated the effects of sesamin on TBI in a rat model. Sesamin, a lignan, reduced neuronal loss and apoptosis while reducing oxidative stress. However, it was shown that sesamin at 30 mg/kg could reduce brain edema as assessed by brain water content. Sesamin was also shown to reduce the loss of tight junction proteins such as occludin. Finally, sesamin was shown to exert its beneficial effects by reducing the activation of ERK, p-38, and caspase-3 [101].

### Magnolol

*Magnolia officinalis* is well known for its root and bark, which contains magnolol, a phenolic compound. Chinese and Japanese medicine have traditionally used this herb to treat a variety of ailments without causing significant toxicity. In addition, magnolol inhibits lipid peroxidation in rat heart mitochondria 1000 times more effectively than tocopherol and 50,000 times better than glutathione [102]. After TBI, magolol (2 mg/kg intravenously) decreased the presence of TBI markers like glycerol and 2,3-dihydroxybenzoic acid, decreased the size of the infarct, and reduced neuronal apoptosis by promoting proteins such as transforming growth factor-beta 1 (TGF-β1) in neurons [103].

### Honokiol

Several species of Magnolia plants contain an abundant amount of lignan called honokiol. This substance has been used traditionally in medicine for centuries. Magnolias are widely distributed worldwide, with most species found in East and Southeast Asia [104]. Honokiol has been identified as a compound with multiple therapeutic functions, including anticancer, antimicrobial, anti-inflammatory, antithrombotic, antidepressant, and neuroprotective effects due to its BBB-crossing properties [105]. Honokiol ameliorated TBI injury in a rat model by reducing inflammation and improving endothelial cell damage. This phytochemical induced vascular endothelial growth factor expression and reduced pyramidal neuron apoptosis [106]. In another study by Wang et al. in 2014, honokiol (0.5 or 2 mg/kg) showed improved motor, sensory and cognitive functions after TBI in a rat model. The compound reduced neuronal apoptosis and lesion size and decreased cell cycle proteins such as cyclin D1, cyclin-dependent kinase 4, retinoblastoma protein, and E2 promoter binding factor 1 [107].

### Cinnamon

As well as being an essential ingredient of cooking, cinnamon (*Cinnamomum cassia*) is also used in traditional medicine to treat gastritis, dyspepsia, inflammatory conditions, and circulatory issues. Various pharmacological properties of cinnamon include anti-inflammatory, anti-diabetic, antimicrobial, and anti-tumor properties [108, 109]. Yulug et al. in 2018 investigated the beneficial effects of cinnamon on TBI in a rat model. Cinnamon (10 mg/kg) reduced lesion size and neuronal apoptosis and may improve neuronal survival. This compound exerts its therapeutic effects by suppressing the expression of IL-1, IL-6, and NF-κb expression. Cinnamon regulated the expression of various factors such as MDA, CAT, SOD, and GSH. Cinnamon reduced brain edema, as assessed by brain water content [110]. In another in vivo study, Cinnamon was shown to reduce iNOS expression in an animal model after TBI. This compound reduced the activation of microglia and astrocytes. And vascular damage and lessen the size of the lesion while improving memory and motor ability [111].

## Conclusion and Future Perspectives

Traumatic Brain Injury (TBI) stands as a significant public health concern, constituting a leading cause of disability, particularly affecting individuals under 45 years old. TBI encompasses primary and secondary injuries, often accompanied by systemic symptoms. Secondary injury arises from a complex cascade of oxidative and apoptotic events following head trauma, underscoring the critical role of oxidative stress in TBI's injury pathways and potential therapeutic implications. While several studies, such as the one by Zahedi et al., suggest promising effects of curcuminoids, developing effective therapeutic strategies necessitates rigorous preclinical studies and clinical trials. The heterogeneous nature of TBI underscores the need for comprehensive research addressing translational barriers. Several phytochemicals, including QUE, RSV, cinnamon, honokiol, and magnolol, have demonstrated the capacity to modulate signaling pathways crucial in attenuating neuroinflammation associated with TBI. For instance, they have shown inhibitory effects on pathways like TLR4, NF-κB, and NLRP-3 while generally upregulating Nrf2 and PI3K/AKT signaling pathways. These phytochemicals also exhibit modulation of various inflammatory and pro-inflammatory cytokines (e.g., IL-13, IL-1β, IL-6, IL-18, IL-15, TNF-α), collectively underscoring their neuroprotective potential in TBI. However, despite these promising findings, the absence of robust clinical trials poses a significant gap. It is imperative to design clinical trials that assess the appropriate dosage, safety, bioavailability, pharmacokinetics, duration of use, sustained improvement, and potential side effects of phytochemicals, in particular polyphenols, in patients suffering from TBI. Furthermore, elucidating the specific signaling pathways, molecules, and genetic components involved in the therapeutic effects of phytochemicals in TBI models remains an essential avenue for research to better understand their mechanisms of action.

## Data Availability

This is a review article and there is no associated primary data.

## Abbreviations

TBI: Traumatic brain injury
WHO: World Health Organization
BACE-1: β-Amyloid precursor protein–cleaving enzyme 1
NSAIDs: Non-steroidal anti-inflammatory agents
NF-κB: Nuclear factor-kappa β
AD: Alzheimer’s disease
PD: Parkinson’s disease
RNS: Reactive nitrogen species
ROS: Reactive oxygen species
BBB: Blood-brain barrier
RSV: Resveratrol
PQQ: Pyrroloquinoline quinone
GSK-3β: Glycogen synthase kinase 3 beta
NF-κB: Nuclear factor kappa B
TLR4: Toll-like receptor 4
IL-1β: Interleukin-1β
NLRP3: NLR family pyrin domain containing 3
TNF-α: Tumor necrosis factor-α
iNOS: Inducible nitric oxide synthase
MDA: Malondialdehyde
8-oHdG: 8-Hydroxy-2'-deoxyguanosine
SOD: Superoxide dismutase
GSH: Glutathione
CAT: Catalase
Nrf2/HO-1: Nuclear factor erythroid 2 related factor 2/heme oxygenase 1
Nrf2: Nuclear factor erythroid 2 related factor 2
HO-1: Heme oxygenase-1
PI3K/AKT: Phosphatidylinositol 3-kinase/protein kinase B
Bax: BCL2-associated X
Bcl-2: B-cell lymphoma 2
ERK 1/2: Extracellular signal-regulated kinase
PPAR-γ: Peroxisome proliferator-activated receptor-γ
PGC-1α: PPAR-γ coactivator-1α
MMP: Mitochondrial membrane potential
EGCG: Epigallocatechin-3-gallate
IKK α/β: I kappa B kinase alpha/beta
TGF-1β: Transforming growth factor beta 1
PAMPs: Pathogen-associated molecular patterns
LPS: Lipopolysaccharide
Myd88: Myeloid differentiation factor 88
TRAF6: TNF receptor-associated factor 6
TRIF: TIR domain-containing adaptor-inducing interferons
NQO1: Nicotinamide adenine dinucleotide phosphate: quinone oxidoreductase-1
GSH-Px: Glutathione peroxidase
ARE: Antioxidant response element
CNS: Central nervous system
SD: Sprague Dawley
WS: Wistar
SOD: Superoxide dismutase
CAT: Catalase
MDA: Malondialdehyde
EPO: Erythropoietin
ERK1/2: Extracellular signal-regulated kinase 1/2
PI3K-AKT: Phosphatidylinositol-3-kinase and protein kinase B
PGC1a: Peroxisome proliferator-activated receptor-gamma coactivator
DMSO: Dimethyl sulfoxide
Nrf2: Nuclear factor-erythroid factor 2-related factor 2
AMPK: AMP-activated protein kinase
IκBα: Nuclear factor of kappa light polypeptide gene enhancer in B-cells inhibitor, alpha
NSCs: Neural stem cells
EGCG: Epigallocatechin gallate
TGF-β: Transforming growth factor-β
NF-Κb: Nuclear factor kappa-light-chain-enhancer of activated B cells
GFAP: Glial fibrillary acidic protein
iNOS: Inducible nitric oxide synthase
CCI: Controlled cortical impact
AICAR: 5-Aminoimidazole-4-carboxamide ribonucleotide
CMC: Sodium carboxymethylcellulose
ECs: Endothelial cells
Nrf2/HO-1: Nuclear factor erythroid 2-related factor 2/heme oxygenase 1
FFW: Feeney’s falling weight
GSH-Px: Glutathione peroxidase
SIRT1: Caspase-1 and sirtuin 1
NLRP3: NLR family pyrin domain containing 3
ROS: Reactive oxygen species
RVS: Resveratrol
GSK-3β: Glycogen synthase kinase-3β
IL-6: Interleukin 6
IL-12: Interleukin 12
8-OHdG: 8-Hydroxy-2'-deoxyguanosine
GFAP: Glial fibrillary acidic protein
AQP4: Aquaporin-4
IGF-1: Insulin-like growth factor 1
CGNs: Cerebellar granular neurons
p38MAPK: P38 mitogen-activated protein kinases
TLR4: Toll-like receptor 4
NF-κB: Nuclear factor-kappa B
MyD88: Myeloid differentiation primary response 88
LPS: Lipopolysaccharide
DMSO: Dimethyl sulfoxide
Cur: Curcumin
IL-1β: Interleukin-1β
RANTES: Regulated on expression normal T-cell expressed and secreted
TNF-α: Tumor necrosis factor-alpha
MCP-1: Monocyte chemoattractant protein-1
IL-6: Interleukin 6
